# Platelet-rich fibrin obtained from different protocols affects the formation of the *in vitro* multispecies subgingival biofilm associated with periodontitis

**DOI:** 10.1080/20002297.2024.2445598

**Published:** 2025-01-01

**Authors:** Fabio Hideaki Uyeda, Gustavo Quilles Vargas, Larissa Matias Malavazi, Tatiane Tiemi Macedo, Aline Paim de Abreu Paulo Gomes, Manuela Rocha Bueno, Pedro Henrique Moreira Paulo Tolentino, Lucas Daylor Aguiar da Silva, Luciene Cristina Figueiredo, Jamil Awad Shibli, Bruno Bueno-Silva

**Affiliations:** a Dental Research Division, Guarulhos University, Guarulhos, Brazil; b Department of Biosciences; Piracicaba School of Dentistry, University of Campinas, Piracicaba, Brazil; c Department of Periodontology, São Leopoldo Mandic Faculty, Campinas, Brazil

**Keywords:** Subgingival biofilm, periodontitis, antimicrobial, platelet-rich fibrin, iPRF

## Abstract

**Background:**

The aim of this article is to evaluate the effect of different portions of Platelet Rich Fibrin (PRF) membranes and liquid-PRF, prepared by two distinct protocols/centrifuges each, on the multispecies subgingival biofilm.

**Materials and methods:**

PRF membranes and liquid-PRF were prepared using two protocols: centrifuge 1 uses fixed acceleration while centrifuge 2, progressive acceleration. PRF samples were introduced into device concurrently with 33-species bacterial inoculum. After seven days, biofilm metabolic activity (MA) and microbial profile were evaluated through colorimetric reaction and DNA–DNA hybridization, respectively.

**Results:**

Among PRF membranes, the ones from centrifuge 1 led to better reduction in MA, total biofilm, and F. periodonticum, P. gingivalis and T. forsythia counts when compared to untreated/centrifuge 2 treated biofilms. However, centrifuge 2 liquid-PRF reduced MA, total biofilm and F. periodonticum counts when compared to untreated/centrifuge 1 treated-biofilms.

**Conclusion:**

PRF membrane and exhibited comparable antibiofilm activity. However, PRF distinct forms, obtained by same centrifugation protocol, may present different antimicrobial properties.

## Introduction

Periodontitis is an inflammatory disease associated with dysbiotic dental biofilm, which invades the subgingival environment and generates a persistent and unbalanced inflammatory response that results in the destruction of the tooth support tissue [1,2]. Its main characteristics include the loss of periodontal tissue, manifested through the loss of clinical attachment level and alveolar bone loss, the presence of periodontal pockets and bleeding on probing (BOP) [3]. Periodontopathogenic biofilm is a complex microbial community consisting of microcolonies that adhere to the tooth surface in both supra- and subgingival environments. Even in clinically healthy sites, individuals with periodontal disease exhibit higher proportions of pathogenic microorganisms compared to those without the disease [4,5].

Initially, periodontal treatment should focus on motivating the patient to improve their oral health through proper hygiene practices, professional plaque removal, and supragingival control [6]. Following this, the focus should shift to controlling the subgingival biofilm. In this stage, the gold standard therapy is scaling and root planning, as it effectively reduces probing depth and bleeding on probing, promotes clinical attachment gain, and alters the microbial profile. Chemical biofilm control may be used at this time as adjuvant treatment [6]. However, this intervention alone may not resolve all cases, and in such instances, surgical therapy is recommended to enable more effective subgingival instrumentation or regenerative treatment. For advanced stages of periodontitis, such as stage IV, an interdisciplinary approach is often required for the rehabilitation of the compromised dentition [6].

Regenerative therapy may be associated with the treatment of residual pockets, with the aim of restoring them. Among the options available, platelet-rich fibrin (PRF) was developed as a source of autologous growth factors derived from the patient’s own blood. In dentistry, PRF has been used in the treatment of post-extraction alveoli, gingival recessions, palatal wound closure, periodontal defect regeneration, and hyperplastic gingival tissue [7] due to the presence of various growth factors, which are released at the site after application. In this way, PRF membranes, prepared by distinct protocols, can be used as an adjunct to procedures to potentially minimize post-operative infection and accelerate wound healing, alone or over a collagen membrane [8].

More recently, some studies have also explored their antibacterial potential [9–11]. The antimicrobial effect of PRF has been demonstrated on red complex bacteria (*Porphyromona gingivalis*, *Treponema denticola*, *Tanerella forsytia*) and also on pathogenic biofilm-forming staphylococcal bacteria isolated from patients with oral abscesses [12]. A systematic review, published in 2023, which included 16 articles, found that only two studies evaluated the effect of PRF on two-species biofilm [13,14], Some of the other studies included in the review collected biofilm directly from patients’ periodontal pockets but did not specifically assess any bacteria [9,15]. Lastly, the remaining studies focused on the effect of PRF on planktonic bacteria [16]. The broad spectrum of inhibitory and bactericidal activity of PRF may be due to its composition of platelets, fibrin, fibronectin, thrombin, HBD3 peptide (antimicrobial peptide), myeloperoxidase and white cells inclusion [13]. Current evidence suggests that platelets may play multiple roles in the host’s antimicrobial defense by generating oxygen metabolites, including superoxide, hydrogen peroxide and hydroxyl-free radicals, capable of binding, aggregating and internalizing microorganisms, as well as releasing a series of potent antimicrobial polypeptides [11].

Studies have evaluated the potential differences between different centrifugation protocols. High-speed centrifugation produces a larger clot and a denser fibrin-like morphology compared to low-speed PRF clots, and a greater number of visible cells were identified at the bottom of the tube, near the red clot. The PRF clots produced by low-speed centrifugation produced smaller clots, with platelets better distributed throughout the PRF clot. Therefore, depending on the centrifugation protocol, platelets may be homogeneously distributed from the superficial surface to the deeper layers or may be more concentrated in the proximal superficial region [17,18]. Based on the above, we selected two centrifuges from different brands, each with distinct recommended protocols: one featuring a variable acceleration speed for centrifugation and the other with a fixed centrifugation velocity in order to evaluate any potential differences in antimicrobial properties based on the centrifugation protocols. In this way, the objective of this article is to evaluate the antimicrobial effect of different portions of platelet-rich fibrin, obtained as membrane and liquid from two centrifuges, on a multispecies biofilm associated with periodontitis.

## Material and methods

This research project was approved by the Human Research Ethics Committee of the University of Guarulhos (number 60379922.20000.5506). Informed consent was obtained from all participants in this study.

***Inclusion criteria:*** Men and women aged 18–65 years; - Good general health (absence of any condition that could pose a risk to the subject during participation in the study. Examples: heart problems, chronic kidney problems, etc.); - Willingness to provide information about their medical history; - At least 15 permanent natural teeth without crowns (excluding third molars).

***Exclusion criteria:*** Use of anticonvulsants, antihistamines, antidepressants, sedatives, tranquilizers, anti-inflammatory drugs, or daily analgesics within one month prior to study entry or scheduled to start such use during the course of the study; - Pregnant or breastfeeding women; - Prolonged use of antibiotics at any time during the three months prior to study entry; - Need for antibiotic prophylaxis; - Continued use of medications known to affect gingival tissue (i.e. periodontitis (purulent exudate, tooth mobility and/or extensive loss of periodontal attachment); probing depth greater than 4 mm; - smokers or those with a history of alcohol or drug abuse; - diabetics; - participants in another clinical trial.

### PRF obtainment

Blood samples were collected from three subjects on the same day as the onset of biofilm formation. Plastic vacuum tubes with no chemical additives inside were used to obtain liquid-PRF and plastic vacuum tubes with silica blasted inside were used to obtain PRF clots. After collection, the tubes were not homogenized or shaken to avoid precoagulation of the sample but were centrifuged immediately after collection. Standardization of platelet-rich fibrin (PRF) studies heavily depends on the precise application of relative centrifugal force (RCF) and rotations per minute (RPM) protocols. RCF is a critical parameter that is influenced by the rotor radius, which can vary across different centrifuge models. RPM, on the other hand, measures the number of revolutions a tube makes per minute. However, RPM alone does not accurately represent the actual force exerted on the contents of the tube, as the force depends on both the speed and the rotor radius. For this reason, RCF offers a more accurate assessment of the centrifugal forces acting within the system [19].

Two different centrifuges with three different protocols were used as follows: centrifuge 1 (DAIKI®) uses RCF 256 × g/5 min centrifugation to obtain liquid-PRF and RCF 448 × g/10 min centrifugation to prepare PRF membrane, while centrifuge 2 (DRILLER®) uses centrifugation with progressive acceleration (RCF 30 × g for 5 min, RCF 104 × g for 5 min and RCF 354 × g rpm for 5 min) to obtain both liquid-PRF and membrane. After centrifugation, the PRF clots were placed in sterile metal boxes specifically designed for dehydration and formation of PRF membranes. After dehydration, fragments of the membranes (deep and superficial portions) were used. There were nine groups in the study, as described below:

– Control: Biofilm without treatment
– MP-2110: deep portion of PRF membrane obtained at RCF 448 × g/10 min (centrifuge 1)
– MS-2110: superficial portion of PRF membrane obtained at RCF 448 × g/10 min (centrifuge 1)
– MP-PROG: deep portion of PRF membrane obtained by progressive acceleration centrifugation (centrifuge 2)
– MS-PROG: superficial portion of PRF membrane obtained by progressive acceleration centrifugation (centrifuge 2)
– LP-1600: deep portion of liquid PRF obtained at RCF 256 × g/5 min (centrifuge 1)
– LS-1600: superficial portion of liquid PRF obtained at RCF 256 × g/5 min (centrifuge 1)
– LP-PROG: deep portion of liquid PRF obtained by progressive acceleration centrifugation (centrifuge 2)
– LS-PROG: superficial portion of liquid PRF obtained by progressive acceleration centrifugation (centrifuge 2)

### Multispecies subgingival biofilm

The following species (with respective ATCC numbers) were used: *Actinomyces naeslundii* (12104), *Actinomyces oralis* (43146), *Actinomyces gerencseriae* (23840), *Actinomyces israelii* (12102), *Veillonella parvula* (10790), *Actinomyces odontolyticus* (17929), *Streptococcus sanguinis* (10556), *Streptococcus oralis* (35037), *Streptococcus intermedius* (27335), *Streptococcus gordonii* (10558), *Streptococcus mitis* (49456), *Aggregatibacter actinomycetemcomitans* (29523), *Capnocytophaga ochracea* (33596), *Capnocytophaga gingivalis* (33624), *Eikenella corrodens* (23834), *Capnocytophaga sputigena* (33612), *Streptococcus constellatus* (27823), *Eubacterium nodatum* (33099), *Fusobacterium nucleatum vincentii* (49256), *Parvimonas micra* (33270), *Fusobacterium nucleatum polymorphum* (10953), *Campylobacter showae* (51146), *Fusobacterium periodonticum* (33693), *Prevotella intermedia* (25611), *Porphyromonas gingivalis* (33277), *Tannerella forsythia* (43037), *Eubacterium saburreum* (33271), *Streptococcus anginosus* (33397), *Selenomonas noxia* (43541), *Propionibacterium acnes* (11827), *Gemella morbillorum* (27824) and *Streptococcus mutans* (25175).

The microorganisms were cultured under anaerobic conditions (85% nitrogen, 10% carbon dioxide, and 5% hydrogen) on tryptone soy agar supplemented with 5% sheep’s blood, with *P. gingivalis* and *P. intermedia* cultured on tryptone soy agar supplemented with yeast extract enriched with 1% hemin, 5% menadione, and 5% sheep’s blood, while *T. forsythia* was cultivated on tryptone soy agar with yeast extract enriched with 1% hemin, 5% menadione and 5% sheep’s blood and 1% N-acetylmuramic acid. After 48 h of growth, all species were transferred to BHI broth (Becton Dickinson, Sparks, MD) supplemented with 1% hemin. Then, after 24 h, the optical density (OD) at 660 nm was adjusted to 0.1, corresponding to approximately 10^8^ cells/ml of each species. The individual cell suspensions of each species were diluted to obtain the final biofilm inoculum containing 10^4^ cells of each bacterial species. The multispecies subgingival biofilm model was developed using the Calgary Biofilm Device (CBD) according to Miranda et al. [20]. Biofilms were grown for 7 days and the medium was changed after 72 h of incubation. On the first day, liquid-PRF and PRF membranes were added to the CBD right after the bacterial inoculum. The PRF samples were prepared immediately before inoculation of the biofilm into the Calgary apparatus, according to the groups described above.

### Biofilm metabolic activity

The percentage reduction in biofilm metabolic activity was determined using 2,3,5-triphenyltetrazolium chloride (TTC) and spectrophotometry. TTC is used to distinguish metabolically active from inactive cells. The white substrate is enzymatically reduced to red 1,3,5-triphenyl formazan (TPHP) by living bacterial cells through the activity of various dehydrogenases. The colour change of the substrate is measured spectrophotometrically to determine the rate of reduction, which is used as an indirect measure of bacterial metabolic activity. To measure the metabolic activity of the biofilms, the rods were washed with wash solution and transferred to plates with 200 µl per well of fresh BHI medium containing 1% hemin and 0.1% TTC solution. The plates were then incubated for 12 h at 37°C under anaerobic conditions. The TTC conversion was read at 485 nm using a spectrophotometer [21].

### DNA-DNA hybridization (checkboard DNA-DNA)

For the microbial profile,10 pins coated with 7-day biofilm from each group were washed twice with PBS and transferred to Eppendorf tubes containing 100 uL TE buffer (Tris 10 mm -HCl, EDTA 1 mm [pH 7.6]), followed by 100 uL 0.5 M NaOH. The tubes containing the pins and the final solution were boiled for 10 min, and the solution was neutralized by adding 0.8 ml of 5 M ammonium acetate. The samples were then individually analyzed for the presence and amount of the 33 bacterial species using the DNA-DNA hybridization technique. Briefly, after lysis of the samples, the DNA was loaded into lanes on a nylon membrane using a minislot device (Immunetics, Cambridge, MA). After the DNA was fixed to the membrane, which was placed in a Miniblotter 45 (Immunetics), digoxigenin-labeled whole-genome DNA probes for the subgingival species used were hybridized to individual lanes of the Miniblotter 45. After hybridization, the membranes were washed, and the DNA probes were detected using an antibody specific for digoxigenin conjugated to alkaline phosphatase. Signals were detected with AttoPhos substrate (Amersham Life Sciences, Arlington Heights, IL) and results were read with Typhoon Trio Plus (Molecular Dynamics, Sunnyvale, CA). Two lanes in each run contain patterns with 10^5^ or 10^6^ cells of each species. Signals obtained with Typhoon Trio were converted to absolute counts by comparison with standards on the same membrane. No signal was recorded as zero [21].

### Statistical analysis

**Figure 1.**
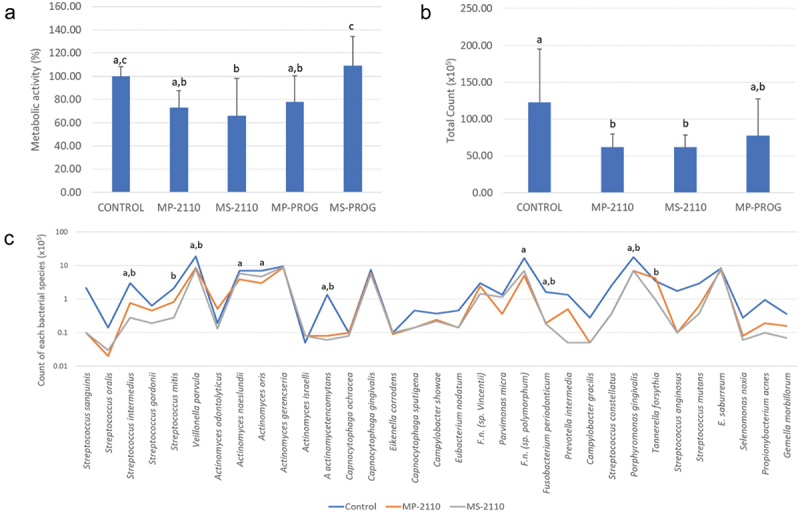
Results of biofilms treated with PRF membranes obtained under different protocols.

**Figure 2.**
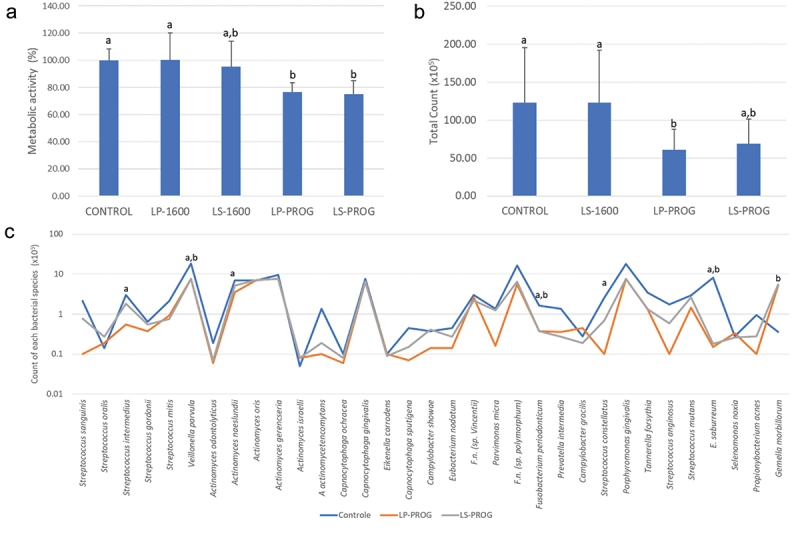
Results of iPRF-treated biofilms obtained under different protocols.

**Figure 3.**
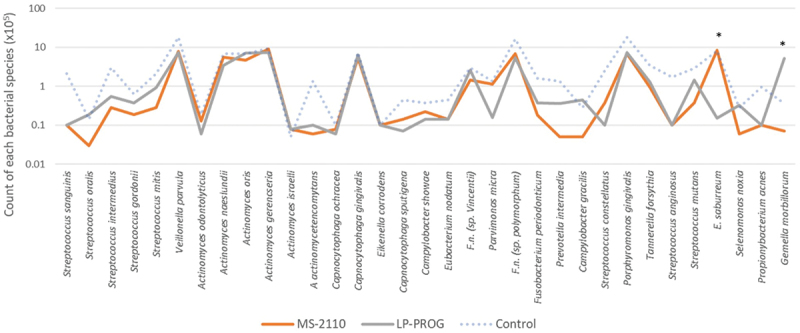
Average counts of each bacterial species in biofilms treated with MS-2110 (surface portion of PRF membrane obtained by centrifugation at 2110 rpm) and LP-PROG (deep portion of iPRF obtained by centrifugation at progressive speed). Statistical analysis by Kruskal-Wallis followed by Dunn (*p* ≤ 0.05). ’*’ indicates statistically significant difference between MS-2110 and LP-PROG. The blue dashed line with transparency represents the data from the control biofilm, which in this specific case was not included in the statistical analysis.

The Biostat Computer Software (Version 5.3) was used for statistical analysis. The results of the metabolic analysis (Figures 1a and 2a) were statistically analyzed by ANOVA followed by Tukey’s test, while the checkerboard data (presented as ‘total counts’ - Figures 1b and 2b - and the “mean counts - Figures 1c, 2c and 3) were analyzed by Kruskal-Wallis test followed by Dunn’s post hoc test with 5% statistical significance.

a: Mean and standard deviation of metabolic activity of biofilms treated with different PRF membrane groups. Different letters indicate statistical difference by ANOVA followed by Tukey’s test (*p* ≤ 0.05);

b: Mean and standard deviation of total biofilm counts treated with different PRF membrane groups. Different letters indicate statistical difference by Kruskal-Wallis followed by Dunn’s test (*p* ≤ 0.05).

c: Average counts of each bacterial species in the control biofilms and those treated with MP-2110 (deep portion of PRF membrane obtained by centrifugation at 2110 rpm) and MS-2110 (superficial portion of PRF membrane obtained by centrifugation at 2110 rpm). Statistical analysis by Kruskal-Wallis was followed by Dunn (*p* ≤ 0.05). Letter ‘a’ indicates statistically significant difference between the control group and MP-2110, letter ‘b’ indicates statistically significant difference between the control group and MS-2110.

a: Mean and standard deviation of metabolic activity of biofilms treated with different iPRF groups. Different letters indicate statistical difference by ANOVA followed by Tukey’s test (*p* ≤ 0.05);

b: Mean and standard deviation of total biofilm counts treated with different iPRF groups. Different letters indicate statistical difference by Kruskal-Wallis followed by Dunn’s test (*p* ≤ 0.05).

c: Average counts of each bacterial species in the control biofilms and those treated with LP-PROG (deep part of the iPRF obtained by centrifugation at progressive speed) and LS-PROG (superficial part of the iPRF obtained by centrifugation at progressive speed). Statistical analysis by Kruskal-Wallis was followed by Dunn (*p* ≤ 0.05). Letter ‘a’ indicates statistically significant difference between control and LP-PROG, letter ‘b’ indicates statistically significant difference between control and LS-PROG.

## Results

First, only PRF membrane groups were compared against each other and next, the same comparison was made for liquid PRF samples to determine which centrifuge/protocol in a specific form (membrane or liquid) had the best anti-biofilm activity. In this sense, Figure 1a shows the metabolic activity (MA) results of the control and PRF membrane biofilm treated groups. The MS-2110 (superficial portion PRF membrane obtained by RCF 448 × g/10 min) was the only group that showed a statistically significant lower MA compared to the control group (*p* ≤ 0.05). The MP-2110 (deep portion of PRF membrane obtained at 2110 rpms/10 min) and MP-PROG (deep portion of PRF membrane obtained by progressive acceleration) groups did not show statistical significance (*p* ≥ 0.05) when compared to the control or MS-2110 groups. The MS-PROG group did not differ from the control group (*p* ≥ 0.05) and had a higher mean metabolic activity than the other test groups (*p* ≤ 0.05). Therefore, due to higher MA values, this group was not used in subsequent analyses.

Figure 1b shows the total bacterial count data. The MP-2110 and MS-2110 groups showed ~50% less total counts than the control (*p* ≤ 0.05). The MP-PROG group did not show a significant difference from any of the other groups (control or treatments) and was therefore removed from the next analysis. Figure 1c shows the mean counts of each bacterial species. The MP-2110 group reduced the count of 8 species while the MS-2110 group reduced the count of 7 species compared to the control group (*p* ≤ 0.05). Notably, both PRF membrane treatments reduced the counts of *P. gingivalis* and *F. periodonticum* and only the MS-2110 group reduced the counts of *T. forsythia* (*p* ≤ 0.05).

Figure 2a shows MA results of biofilms treated with groups LP-1600 (deep portion of liquid-PRF obtained by centrifugation at RCF 256 × g/5 min), LS-1600 (superficial portion of liquid-PRF from the same centrifugation protocol), and with the deep and superficial portions of liquid-PRF prepared by centrifugation with progressive acceleration (groups LP-PROG and LS-PROG, respectively). Biofilms treated with LP-1600 and LS-1600 were not statistically different from untreated biofilms (*p* ≥ 0.05), while biofilms treated with LP-PROG and LS-PROG showed a significant 20% reduction in MA compared to the control (*p* ≤ 0.05). As the results of the LP-1600 group were statistically higher (*p* ≤ 0.05) than the values of the two groups with the best activity (LP-PROG and LS-PROG) and did not differ from the control group (*p* ≥ 0.05), this group was excluded from further analyses.

Results for total biofilm counts are shown in Figure 2b. Treatment with LP-PROG reduced total biofilm counts by ~40% compared to control (*p* ≤ 0.05). The LS-PROG group was not statistically different from any of the other groups (*p* ≥ 0.05). To maintain the pattern adopted with the membranes (Figure 1), it was decided to exclude only LS-1600 at this point because the results were similar to the control group (*p* ≥ 0.05) and different from the LP-PROG group, with better activity (*p* ≤ 0.05).

Figure 2c shows the mean counts of each bacterial species present in the biofilms. The LP-PROG treatment reduced the counts of 6 bacterial species compared to the control, while the LS-PROG treatment reduced the counts of 3 species and increased the counts of *G. morbillorum* compared to the control (*p* ≤ 0.05). Of particular note is the reduction of *F. periodonticum* by both treatments’ groups (*p* ≤ 0.05). On the other hand, none of the treatments were able to reduce the counts of *P. gingivalis* and *T. forsythia* (*p* ≥ 0.05), members of the red complex.

Next, we compared the best membrane and liquid groups. In this sense and according to the number of reduced species and also, the pathogenicity of such species, we chose to compare data from MS-2110 treatment (due to its effect on *T. forsithya*) and LP-PROG (due to the greater number of reduced species). The transparent blue dashed line in Figure 3 represents the values of the control group and was included in the graph only to illustrate the values of each species but was not included in the statistical analysis as this had already been done.

The only bacterial species that showed statistical significance between the MS-2110 and LP-PROG treated biofilms were *E. saburreum* and *G. morbillorum*, with the MS-2110 treated biofilm showing higher *E. saburreum* counts while the LP-PROG treated biofilm showed increased *G. morbillorum* counts. The proximity of the lines from both groups is striking, indicating a very similar effect on the counts of the different species contained in the biofilm.

## Discussion

The present study compared the effectiveness of both PRFs forms (membrane and liquid) obtained from two different centrifuges in their ability to combat bacterial biofilms. In addition, deep and superficial portions of both PRF forms were also evaluated. The results showed that among the all portions of all PRF membranes obtained from the two centrifuges tested, the MS-2110 group, corresponding to the upper part (superficial part) of the PRF membrane obtained by centrifugation at 2110 rpm for 10 min (centrifuge 1), showed better results in all parameters evaluated (metabolic activity, total biofilm counts and mean counts of specific bacterial species). Meanwhile, the LP-PROG group, representing the lower (deep) part of the liquid PRF prepared by centrifugation with progressive acceleration (centrifuge 2), showed better results in the same all evaluated parameters among all portions of every liquid-PRF sample tested. Subsequently, the comparative analysis between the MS-2110 and LP-PROG treatments showed a remarkable similarity with regard to the effects observed on the mean counts of each bacterial species present in the biofilm model.

Among the bacterial species reduced by PRF, *P. gingivalis* and *T. forsythia* stand out. The current concept of periodontitis identifies a dysbiotic microbial community as responsible for the onset of the disease, characterized by an exacerbated inflammatory response [22]. Thus, members of the red complex still play a crucial role in the development of the disease. Among them, *P. gingivalis* and *T. forsythia* are the most studied microorganisms. Both have been proposed as targets for the prevention of oral microbiome dysbiosis, as their presence may contribute to the shift from ‘healthy’ to disease-associated biofilm [23]. *P. gingivalis* and *T. forsythia* are species that orchestrate microbial dysbiosis and contribute to the mechanisms by which microorganisms evade the human immune system.

In recent years, the virulence factors of *P. gingivalis* have been well studied because of their pathogenicity. Among its virulence factors, gingipains can subvert the human immune system, allowing the establishment of a dysbiotic subgingival microbiota. FimA, a fimbrial protein that inhibits the complement system receptor on macrophages, is another notable virulence factor produced by *P. gingivalis*.

*T*. *forsythia* is also a known periodontopathogen. Among its virulence factors, the BspA protein appears to be associated with increased alveolar bone loss during periodontitis. The metabolic products of its peptidoglycan degradation may also contribute to undermining the host immune system and promoting the formation of a dysbiotic subgingival biofilm. The presence of a pathogenic microbiota leads to an exacerbated inflammatory response that ultimately results in the destruction of the supporting periodontal tissues, with progressive clinical attachment loss and ultimately tooth loss. In addition to its involvement in periodontitis, *T. forsythia* may also play a significant role in the pathogenesis of peri-implantitis. This microorganism was found in elevated levels at dental implant replacements than in the adjacent tooth, and its presence is associated with an increased severity of peri-implantitis [24–26].

*F. periodonticum* is another bacterium affected by both forms of PRF, liquid and membrane. The Fusobacterium genus plays a crucial role in the transition from periodontal health to disease. Serving as an intermediate colonizer within dental biofilms, Fusobacterium species are among the first Gram-negative organisms to establish a stable presence in the subgingival biofilm. This pivotal role involves facilitating interactions between Gram-positive and Gram-negative species, thereby promoting the colonization of additional anaerobic species, including those recognized as pathogens within the red complex [27]. Among the Fusobacterium species, *F. periodonticum* has recently been associated with oral malodor, being one of the species with higher abundance in individuals suffering from oral malodor and contributing significantly to sulfur metabolism [28]. This microorganism is also found in higher numbers in individuals with oral squamous cell carcinoma [29,30]. Therefore, the effects of PRF on *F. periodonticum* could be a noteworthy discovery with implications beyond dentistry.

Thus, future studies should evaluate the mechanism by which PRF inhibits the presence of *P. gingivalis*, *T. forsythia* and *F. periodonticum* in the biofilm and whether there is a possible effect of PRF on the virulence factors of the two pathogens. Regarding the antimicrobial mechanisms of PRF, the literature showed that pre-treatment of PRF exudate with HRP (peroxidase inhibitor) eliminated its antimicrobial properties, which was not the case when PRF was pre-treated with trypsin (protease inhibitor). Therefore, that study conclude that the anti-biofilm effect of PRF may be due to the action of peroxides [31]. In addition, a recent study demonstrated that both the membrane and liquid forms of PRF obtained from patients with periodontitis showed better inhibition of *A. actinomycetemcomitans* monospecies biofilm formation than PRF obtained from patients without periodontitis [31]. Thus, future studies may test these mechanisms using robust models such as those performed here.

The majority of tubes utilized to obtain the PRF membrane are coated with silica, as the plastic surface lacks the capacity to effectively stimulate coagulation when used alone [32]. Silica exists in two principal forms: crystalline and amorphous. The crystalline form is widely distributed in nature, occurring in minerals such as quartz and in sand. Prolonged exposure to these particles, which is common in activities such as mining, can result in chronic diseases such as silicosis, an irreversible inflammatory condition of the lungs [33,34]. In contrast, amorphous silica is less toxic and considered safer, with minimal chronic effects. Consequently, the amorphous variety is extensively utilized in the pharmaceutical sector, exemplified by its incorporation in PRF tubing. Moreover, it has been approved by the Food and Drug Administration as a food additive [35]. However, recent studies indicate that amorphous silica may induce inflammation or cytotoxicity, raising concerns about potential interactions at subclinical levels with unknown factors that could pose unexpected health risks. In light of this, recent research has developed a method that, rather than using silica, employs a synthetic protein inside a tube to indirectly stimulate coagulation. However, this plastic-modified tube is not currently available on the market [32].

Platelet-rich fibrin can be prepared using different protocols, which may affect the mechanical and bioactive properties of PRF. Advances in centrifugation techniques, such as horizontal centrifugation and customized Platelet-Rich Fibrin (C-PRF), have enhanced the utility and efficacy of PRF in regenerative applications. Horizontal centrifugation enables uniform and efficient separation of cell layers, minimizing cell accumulation at the tube ends and promoting greater retention of leukocytes and platelets within the fibrin matrix. This process increases the concentration of immune cells crucial for PRF’s regenerative properties. C-PRF further refines the centrifugation process by tailoring parameters such as speed, duration, and rotor radius to individual patient characteristics, including blood volume and clinical conditions. This personalized approach optimizes the biological efficacy of PRF by ensuring an ideal concentration of immune cells, platelets, growth factors, and other bioactive components within the fibrin matrix [36].

In 2023, a study evaluated three centrifugation protocols, where protocol A centrifuged blood at 1300 rpm for 8 min, protocol B centrifuged at 2300 rpm for 12 min, and protocol C centrifuged at 1500 rpm for 14 min. The results showed that protocol B produced the largest zones of inhibition [37]. This is corroborated by the present manuscript since the protocol with 2100 rpm centrifugation also presented the best results. It is important to realize that the scientific evidence supporting the clinical treatment of periodontitis is largely based on randomized clinical trials and systematic reviews. Therefore, while the present results do not yet provide conclusive support for the clinical use of PRF solely for antimicrobial purposes, there is ample evidence supporting its clinical use for other objectives such as periodontal healing and bone regeneration [38–41]. Hence, the present antimicrobial study could be considered as the initial step in the process, potentially paving the way for future *in vivo* studies aiming to further explore the anti-biofilm effects of PRF since the previous literature evidence (until the present study) related to PRF antimicrobial property was formed by only of monospecies biofilms *in vitro* studies [9,13–15]

In addition, the present research may suggest a new adjunctive treatment modality for residual periodontal pockets, since even after mechanical periodontal treatment, not all bacteria in the pocket are eliminated as they invade the tissues. Therefore, areas with deep or complex defects, such as infra-osseous lesions and furcation regions, are often areas where periodontitis recur. Accordingly, strategies such as antimicrobials or immunomodulators may promote healing of these areas. Another related clinical situation in which the use of adjuvants may be beneficial is the treatment of peri-implantitis, as *T. forsythia* has been found at elevated levels in dental implant replacements as mentioned above and PRF presents strong antimicrobial effect on it as demonstrated here. One more possibility for the clinical use of PRF with antimicrobial purposes, but in periodontally healthy patients, would be in cases of implants where the success of immediate osseointegration depends on a region with minimal or no infection. In this way, an associated strategy that interferes with bacterial colonization in the inflammatory phase of osseointegration is also very interesting [24–26]. However, it is important to emphasize that the efficacy of this potential therapy for residual (or recurrent) pockets and prevention of dental implants infection (whether in patients with periodontal disease or in healthy patients) also needs to be evaluated in future clinical trials.

Furthermore, our experimental design did not allow us to assess whether PRF could effectively target mature biofilms typically encountered in periodontitis patients, as the PRF was applied almost simultaneously with the bacterial inoculum. However, the objective of the present investigation was not related to mature biofilms since we suggest PRF as an antimicrobial treatment for specific recurrent pockets and/or as an adjuvant during the surgical phase of periodontal treatment. Consequently, future studies investigating the potential *in vivo* application of PRF may benefit from incorporating scaling and root planning alongside PRF usage in residual pockets following initial therapy. Another limitation of our study pertains to the semi-quantitative nature and detection limit of the checkerboard assay. However, this methodology enables the simultaneous evaluation of multiple species of microorganisms across numerous samples, and for *in vitro* studies where bacterial species are known prior to results, this methodology should be adequate. In studies involving a smaller number of bacterial species, qPCR may be a suitable option due to its low detection limit and quantitative capabilities. In contrast, DNA–DNA hybridization has traditionally been a commonly used technique in clinical trials, while next-generation sequencing has recently emerged as a valuable tool for microbiological assays. An additional enhancement of this model could involve further analysis techniques, such as confocal microscopy, which would allow for a detailed evaluation of its structure, biomass, and exopolysaccharide content. While confocal microscopy is commonly used to study caries-related monospecies biofilms, its application to periodontal biofilms remains limited. Therefore, future research should focus on expanding our understanding by developing and testing specific dyes for confocal analysis of multispecies biofilms related to periodontitis.

## Conclusion

Platelet-rich fibrin (PRF) in its two presentations (membrane and liquid) showed strong antimicrobial activity against an *in vitro* subgingival biofilm. However, the centrifugation protocol influences the quality of PRF antimicrobial properties. Future studies should evaluate the differences in PRF composition according to the different centrifugation protocols, and their antimicrobial effects should be verified by *in vivo* studies.

## Data Availability

Data are available on request from the authors.
